# Bone-forming peptide-2 derived from BMP-7 enhances osteoblast differentiation from multipotent bone marrow stromal cells and bone formation

**DOI:** 10.1038/emm.2017.40

**Published:** 2017-05-12

**Authors:** Hyung Keun Kim, Jun Sik Lee, Ji Hyun Kim, Jong Keun Seon, Kyung Soon Park, Myung Ho Jeong, Taek Rim Yoon

**Affiliations:** 1 Cardiovascular Convergence Research Center of Chonnam National University Hospital, Gwangju, Korea; 2 Department of Orthopedics Surgery, Center for Joint Disease of Chonnam National University Hwasun Hospital, Jeonnam, Korea; 3 Department of Biology, Immunology Research Lab, BK21-plus Research Team for Bioactive Control Technology, College of Natural Sciences, Chosun University, Gwangju, Korea

## Abstract

Strategies for efficient osteogenic differentiation and bone formation from stem cells would have clinical applications in treating nonunion fracture healing. Many researchers have attempted to develop adjuvants as specific stimulators of bone formation for therapeutic use in patients with bone resorption. Therefore, development of specific stimulators of bone formation has therapeutic significance in the treatment of osteoporosis. To date, investigations of the mature forms of bone morphogenetic proteins (BMPs) have focused on regulation of bone generation. However, we previously identified new peptides from the immature precursor of BMP, and further analysis of these proteins should be performed. In this study, we identified a new peptide called bone-forming peptide-2 (BFP-2), which has stronger osteogenic differentiation-promoting activity than BMP-7. BFP-2 treatment of multipotent bone marrow stromal cells (BMSCs) induced expression of active alkaline phosphatase. In addition, BFP-2 enhanced CD44 and CD51 expression levels and increased Ca^2+^ content in BMSCs. Moreover, radiography at 8 weeks revealed that animals that had received transplants of BFP-2-treated BMSCs showed substantially increased bone formation compared with animals that had received BMSCs treated with BMP-7. Our findings indicate that BFP-2 may be useful in the development of adjuvant therapies for bone-related diseases.

## Introduction

Tissue engineering holds great clinical promise for the repair of segmental bone defects in which bone substitutes are required, such as following removal of infected tissue and bone tumors.^1, 2^ Bone regeneration is one of the most important issues in regenerative medicine and age-related healthcare.^3^ Current drugs that inhibit bone resorption are unsatisfactory; hence, development of bone anabolic molecules is necessary for use in patients who have suffered substantial bone loss. There is a critical need to develop a biomaterial that can chemically and structurally mimic the native extracellular matrix for bone tissue engineering. The enhancement of bone formation is highly important in scaffold-based tissue engineering.

Bone morphogenetic proteins (BMPs) play an important role in regulating cell differentiation and proliferation during development.^4^ They have also been shown to play an important role in stem cell biology. BMPs are the most potent osteo-inductive growth factors, are expressed in many different cells and tissues and were originally investigated due to their ability to regulate new bone formation.^5^ Genetic disruption of BMP genes results in various extraskeletal and skeletal abnormalities during development.^6^ BMP signaling occurs via interaction with two transmembrane serine/threonine kinase receptors, the type I and type II receptors. Activated receptor kinases phosphorylate the transcription factors Smad 1, 5 or 8, which in turn form a heterodimeric complex with nuclear Smad 4 and regulate the expression of target genes in concert with other coactivators.^7, 8, 9, 10^

Most biologically active BMP peptides identified to date are derived from the mature BMP-7 molecule. However, we reported that bone-forming peptide (BFP)-1, which was isolated from the immature precursor of BMP-7, induced osteogenesis and bone formation. We isolated new peptide sequences with osteogenic activity from the immature region of BMP-7. Interestingly, we found that one of the peptide sequences had greater osteogenic activity than mature BMP-7 and induced osteogenesis. We called this peptide BFP-2. This finding offered new insight into the osteogenic activity of BFP-2 and its effect on osteoblasts and further indicated that peptides from the immature region of BMP-7 may be useful in the development of adjuvant therapies for bone-related diseases.

## Materials and methods

### Synthesis and purification of BFP-2

Peptides were synthesized by Fmoc solid-phase peptide synthesis using an ASP48S automated peptide synthesizer (Peptron, Daejon, South Korea) and purified by reverse-phase high-performance liquid chromatography using a Vydac Everest C18 column (250 mm × 22 mm, 10 μm). Elution was carried out with a water-acetonitrile linear gradient (3–40% (v/v) acetonitrile) containing 0.1% (v/v) trifluoroacetic acid. The molecular mass of the purified peptide was confirmed by liquid chromatography/mass spectroscopy using an Agilent (Santa Clara, CA, USA) HP1100 series HPLC system.

### Osteogenic differentiation

Multipotent bone marrow stromal cells (BMSCs) were cultured as previously described.^11^ BMSCs were purchased from the American Type Culture Collection (Manassas, VA, USA) and maintained in Dulbecco's modified Eagle's medium containing 10% fetal bovine serum (Life Technologies, Grand Island, NY, USA) and antibiotics (Life Technologies). Cells were seeded at 1 × 10^4^ cells per well and maintained in culture for 3 days in a humidified 5% CO_2_ atmosphere at 37 °C. Experiments were performed after the cells had reached ~80% confluence. The culture medium was changed after 3 days to osteogenic differentiation medium (ODM; Dulbecco's modified Eagle's medium supplemented with 50 μg ml^−1^ ascorbic acid, 10^−8^ M dexamethasone and 10 mM β-glycerophosphate, all from Sigma-Aldrich (St Louis, MO, USA) to induce osteogenic differentiation. After the cells were cultured for another 3 days, one group of cells was cultured in ODM alone and a second group was cultured in ODM plus BFP-2 (0.01, 0.1 or 1 μg ml^−1^) and 1 μg ml^−1^ of BMP-7 (as a positive control). Cells were analyzed 24 and 48 h later.

### Cell viability

Surviving cells were counted using the MTT (3-(4,5-dimethlythiazol-2yl)-2.5-diphenyltetrazolium bromide) method. MTT (20 μl in 7.2 mM phosphate-buffered saline (PBS), pH 6.5) was added to each well, and the plates were incubated for an additional 3 h. The solution was removed from the wells, and dimethylsulfoxide was added to dissolve the formazan products. The plates were shaken for 5 min, and the absorbance at 570 nm of the solution in each well was recorded on a microplate spectrophotometer.

### Alizarin red S staining

Calcium deposits in cells were quantified as described by Chen.^12^ Briefly, cell cultures were washed twice with distilled water, fixed for 1 h in ice-cold 70% ethanol and rinsed twice with deionized water. Cultures were stained for 10 min with Alizarin red S, and excess dye was removed by gently flushing with running water. Calcium deposits, which appeared bright red, were identified by light microscopy and photographed. Osteogenic differentiation was quantified by determining the density and area of Alizarin red S-stained regions with an image analysis program (Multi GaugeV3.0, Fujifilm, Tokyo, Japan).

### Calcium assays

The calcium content was measured as described by Petros.^13^ Calcium was assayed with a QuantiChrom Calcium Assay kit (Gentaur, Voortstraat, Belgium). Calcium concentration was determined based on the formation of a stable blue complex between phenolsulfonphthalein dye and free calcium, with color intensity directly proportional to the concentration of free calcium in the sample. Color intensity was measured at 612 nm using an Infinite M200 microplate reader (Tecan, Männedorf, Switzerland).

### Reverse transcription-PCR

Reverse transcription-PCR (RT-PCR) was carried out to assess the effects of BFP-2 on the transcription of the genes encoding alkaline phosphatase (ALP) and the internal control housekeeping enzyme glyceraldehyde-3-phosphate dehydrogenase. The primers used were as follows: ALP, (forward) 5′-ACACCTTGACTGTGGTTACTGCTG A-3′ and (reverse) 5′-CCTTGTAGCCAGGCCCGTTA-3′) and glyceraldehyde-3-phosphate dehydrogenase, (forward) 5′-AAATGGTGAAGGTCGGTGTG-3′ and (reverse) 5′-TGAAGGGGTCGTTGATGG-3′. BMSCs grown to 70% confluence on plates in the presence and absence of BFP-2 were homogenized in TRIzol reagent (Life Technologies). Total RNA was isolated, and 0.5 μg aliquots were reverse-transcribed in 20 μl 5 × AMV RT reaction buffer; 2.5 μM poly dT; 1 mM each dATP, dCTP, dGTP and dTTP; 20 U RNAse inhibitor; and 20 U AMV reverse transcriptase. RT was performed with an initial incubation at room temperature for 10 min followed by 42 °C for 15 min, 97 °C for 5 min and 5 °C for 5 min in a GeneAmp PCR System 2700 (Applied Biosystems, Foster City, CA, USA). Aliquots of complementary DNA were amplified using AccuPower GreenStar qPCR premix (Bioneer Co., Daejeon, South Korea) with a Gene Atlas G02 gradient thermal cycler system (Astec, Fukuoka, Japan).

### Immunofluorescence analysis

BMSCs grown on coverslips were fixed with 4% paraformaldehyde in PBS for 15 min, permeabilized with 0.1% Triton X-100 for15 min and then blocked with 5% bovine serum albumin in PBS for 30 min. Coverslips were then incubated with primary antibodies against mouse ALP, osteocalcin and CD44 (eBioscience, San Diego, CA, USA) at a dilution of 1:100 and then with secondary antibodies at a dilution of 1:200; both incubations were at room temperature for 1 h. Cells were washed with PBS, and nuclei were counterstained with 4′,6-diamidino-2-phenylindole. Coverslips were mounted in 70% glycerol, and micrographs were obtained with an Olympus BX50 fluorescence microscope (Tokyo, Japan).

### Flow cytometric analysis

BMSCs (5 × 10^4^) were incubated in staining buffer (PBS containing 0.5% fetal bovine serum and 0.1% sodium azide) containing anti-CD44, anti-CD51 and anti-CD45 (eBioscience) antibodies for 30 min on ice. Cells stained with the appropriate isotype-matched Ig were used as negative controls. After staining, cells were fixed with 2% paraformaldehyde and analyzed with a FACS Calibur instrument equipped with CellQuest software (BD Biosciences, San Diego, CA, USA).

### Cell transplantation

Osteogenically differentiated BFP-2-treated and BMP-7-treated BMSCs were suspended in Dulbecco's modified Eagle's medium at a concentration of 1 × 10^6^ cells per 200 μl. Cells were implanted subcutaneously into the right flank of 6-week-old male C57BL/6 mice and subjected to point projection digital radiography at 26 kV for 3 s using a MX-20 digital microradiography system (Faxitron Bioptics, Lincolnshire, IL, USA). X-ray images were processed using Dicom Works software (LEAD Technologies Inc, Charlotte, NC, USA).

### Histological evaluation

Tissues were fixed in 10% neutral buffered formalin, followed by decalcification in 10% EDTA. Paraffin-embedded samples were sectioned at 8-μm thickness with a microtome. The sections were then floated in a 40 °C water bath, positioned on poly-L-lysine-coated microscope slides and baked for 2 h at 37 °C. The sections were deparaffinized in xylene and rehydrated in a graded ethanol series. Nuclei were stained with hematoxylin for 5 min and then with eosin for 1 min. The sections were covered with permount mounting medium and the coverslips applied. Histological analyses were performed using light microscopy, and the relative osteogenic areas were calculated from at least three randomly selected fields from each specimen using Image-Pro Plus 6.0 software (Media cybernetics, Rockville, MD, USA).

### Statistical analysis

The results are presented as the mean±s.d. The data were analyzed by one-way analysis of variance followed by Duncan's *post hoc* test using SPSS version 11.0 software (Chicago, IL, USA). *P*<0.05 was considered statistically significant.

## Results

### Synthesis of BFP-2

**Figure 1:**
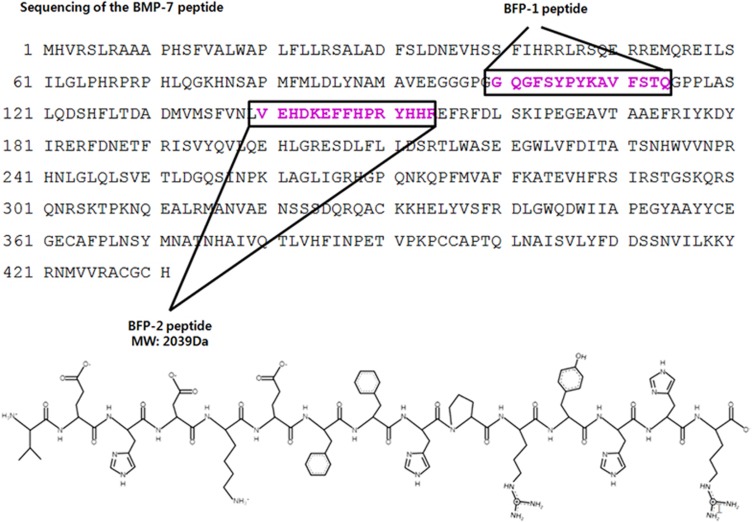
Synthesis of BFP-2. Peptides were synthesized by Fmoc solid-phase peptide synthesis using an automated peptide synthesizer and purified by reverse-phase high-performance liquid chromatography. The molecular masses of the purified peptides were measured by liquid chromatography/mass spectroscopy.

We previously reported the isolation of a new peptide from the immature region of BMP-7 that has osteogenic activity and named the peptide BFP-1. On the basis of these findings, we looked for other new osteogenic peptide sequences in the immature region of BMP-7. In this study, we assessed the osteogenic activity of several peptide sequences derived from the same region of BMP-7. We found that the peptide with the sequence VEHDKEFFHPRYHH, which we called BFP-2, had osteogenic effects in a cell culture system compared with BFP-1. As shown in Figure 1, the complete BFP-2 peptide synthesis was subsequently cloned (GenBank accession number NP_001710).

### BFP-2 induces osteogenic differentiation

**Figure 2:**
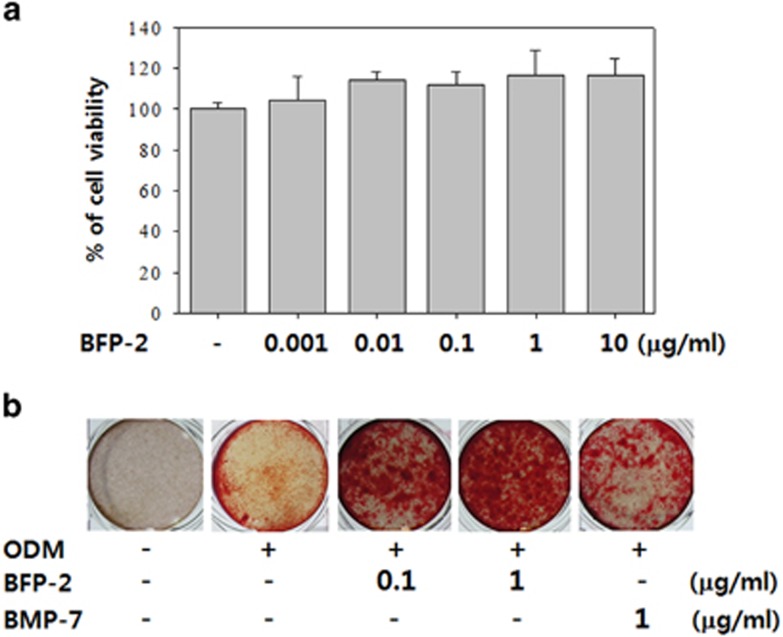
Effect of BFP-2 treatment on osteogenic differentiation. BMSCs were treated with a range of concentrations (0.001–10 μg ml^−1^) of BFP-2 during the initial phase of osteogenic differentiation. Numbers of viable cells were assessed by MTT assays after 24 h incubation (**a**). Cells were treated with 0.1 or 1 μg ml^−1^ of BFP-2 and 1 μg ml^−1^ of BMP-7 and assessed by Alizarin red S staining (**b**). Images are representative of four independent experiments. Magnification, × 20.

Preliminary experiments were performed to determine whether BFP-2 could be used as an adjuvant in osteoporotic disease therapy instead of BMP-7 or BFP-1.We accomplished this by measuring BFP-2-induced osteogenic differentiation in multipotent BMSCs. The possible cytotoxicity of BFP-2 was assessed by treating BMSCs with a range of concentrations of BFP-2 (0.001–10 μg ml^−1^) and measuring cell viability using MTT assays. As shown in Figure 2a, BFP-2 had no cytotoxic effects at these concentrations in BMSCs. BMSCs were treated with 0.1 and 1 μg ml^−1^ of BFP-2 during the initial phase of osteogenic differentiation. As shown in Figure 2b, Alizarin red S staining of the cultures indicated that BFP-2 treatment at this time strongly induced osteogenic differentiation of BMSCs compared with BMP-7 treatment. Moreover, we tested the osteogenic differentiation induced by BFP-2 compared with BFP-1. At the same concentration, BFP-1 and BFP-2 similarly induced osteogenic differentiation of BMSCs (Supplementary Figure 1). These results indicate that BFP-2 might be useful in the development of adjuvant therapies for bone-related diseases.

### BFP-2 induces ALP activity and increasesCa^2+^ concentration

**Figure 3:**
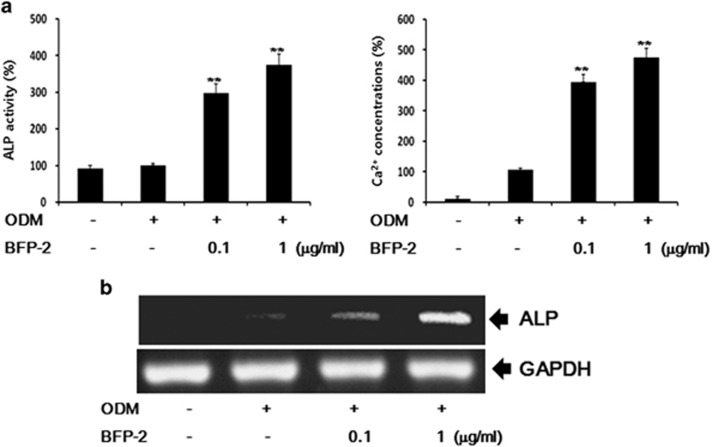
Effect of BFP-2 treatment on ALP activity and Ca^2+^ concentration. BMSCs were treated with 0.1 and 1 μg ml^−1^ of BFP-2 during osteogenic differentiation. ALP activity was quantitatively assayed by measuring the release of *p*-nitrophenyl phosphate with a LabAssay ALP Assay kit. Free Ca^2+^concentration in the cultures was measured with a calcium detection kit (**a**). Total RNA was isolated from control, ODM alone and ODM plus BFP-2-treated cells, and gene expression levels were assayed by RT-PCR (**b**). The data are shown as the mean±s.d. of four independent experiments. **P<0.01 compared with ODM control.

We next assessed whether BFP-2 treatment could enhance the expression of biomarkers of osteogenic activity, ALP activity and Ca^2+^ content. For an optimal concentration of BFP-2, we treated BMSCs with 0.1–10 μg ml^−1^ of BFP-2. Osteogenic differentiation was similar with 1 μg ml^−1^ of BFP-2 and 10 μg ml^−1^ of BFP-2 as shown by Alizarin red S staining (Supplementary Figure 2). As shown in Figure 3a, BFP-2 dose-dependently increased ALP activity and Ca^2+^ concentration in BMSCs. Moreover, we investigated the effects of BFP-2 on the expression of genes involved in osteogenesis. RT-PCR analysis showed that ALP mRNA expression increased during osteogenic differentiation in BFP-2-treated BMSCs compared with BMSCs treated with ODM alone (Figure 3b).

### BFP-2 induces the expression of osteogenic biomarkers

**Figure 4:**
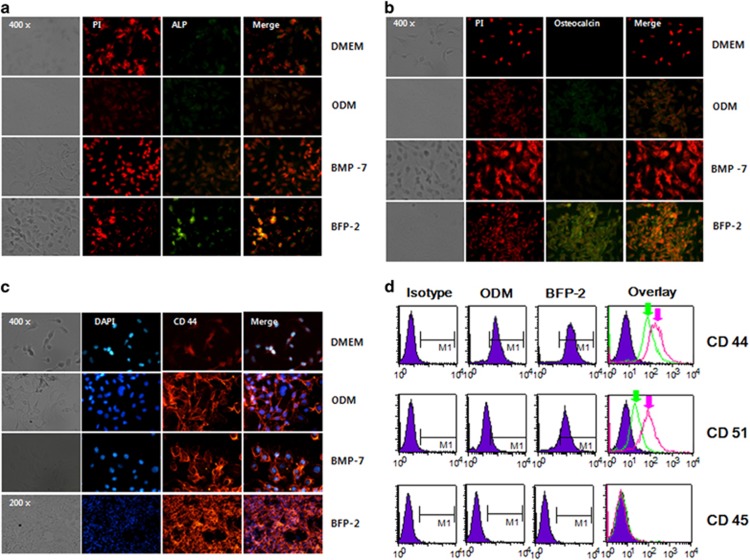
Effect of BFP-2 treatment on ALP, osteocalcin, CD44 and CD51 expression levels. BMSCs were treated with 1 μg ml^−1^ of BFP-2 or1 μg ml^−1^ of BMP-7 (as a positive control). After 24 h, immunofluorescence analyses were carried out using (**a**) anti-ALP (green), (**b**) anti-osteocalcin (green) and (**c**) anti-CD44 (red) antibodies. (**d**) CD44 and CD51 expression was measured by flow cytometry (green arrow: ODM only, pink arrow: ODM plus BFP-2). The data are representative of four independent experiments. Magnification, × 400.

We next investigated whether BFP-2 had more potent effects on osteogenic activity than BMP-7 and whether it could be used as an adjuvant inducer of osteogenesis. Accordingly, we measured BFP-2-induced expression of biomarkers of osteogenic activity, ALP, osteocalcin, CD44, CD51 and CD45, by immunofluorescence staining of the cultures. As shown in Figure 4, BFP-2 treatment significantly induced expression of ALP (Figure 4a) and osteocalcin (Figure 4b), as well as CD44 (Figure 4c), compared with BMP-7 treatment. Interestingly, osteocalcin and CD44 expression levels were more strongly induced by BFP-2 treatment of BMSCs than by BMP-7 treatment. We further investigated the expression levels of CD44, CD51 and CD45 inBFP-2-treated BMSCs by fluorescence-activated cell sorting analysis. We found that BFP-2 induced CD44 and CD51 expression in BMSCs (Figure 4d). These results indicate that the potential osteogenic effects of BFP-2 were through induction of osteocalcin, CD44 and ALP expression.

### BFP-2 induced bone formation

**Figure 5:**
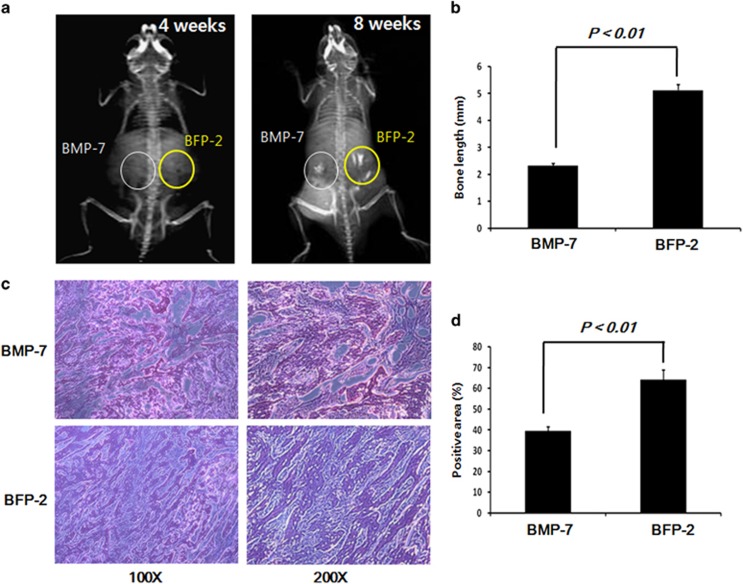
BFP-2 treatment-induced bone formation in an animal model. BMP-7- and BFP-2-treatedBMSCs were injected in the left and right flanks of 6-week-old male mice (*n*=4 per each groups). All mice were examined by radiography at 4 and 8 weeks (**a**). Bone length of BMP-7 and BFP-2 mice at 8 weeks of age (**b**). Bone cells were stained with hematoxylin and eosin (H&E) (**c**) and are shown at magnifications of × 100 or × 200. Quantitative analysis of the osteogenic area on H&E staining images was carried out using the Image-Pro Plus 6.0 software (**d**). The data are representative of four independent experiments.

We next investigated the comparative *in vivo* bone-forming activity of BFP-2 and BMP-7. Radiography analysis 8 weeks after transplantation of cells into animals revealed that those injected with BFP-2-treated BMSCs had significantly increased bone formation compared with those that received BMP-7-treated BMSCs (Figure 5a). We found a larger population of bone cells in the BFP-2-treated group than in the BMP-7-treatedgroup. These results provide new evidence that BFP-2 might be useful as an inducer of osteogenesis, similar to BMP-7 or BFP-1, for bone-related diseases.

## Discussion

Bone is one of the few organs that retains the potential for regeneration into adult life and is the only tissue that can undergo continual remodeling throughout life. Post-fracture bone regeneration progresses through sequential phases similar to endochondral ossification, starting with chemotaxis and proliferation of mesenchymal stem cells. Bone regeneration would have an important impact on the clinical management of various bone and musculoskeletal disorders, such as bone loss and osteoporotic fracture.^14, 15, 16^

BMPs play an important role in regulating cell proliferation and differentiation during development and have also been shown to play an essential role in stem cell biology.^17, 18^ BMPs are known factors in bone regeneration and formation, and many researchers have reported bone-regenerative and other effects of BMPs *in vitro* and *in vivo*. We reported a new peptide sequence derived from BMP-7 as an inducer of osteogenesis and determined the underlying molecular mechanism. Comprehensive analysis of several types of BMP-7-derived peptides led to the earlier finding that BFP-1 derived from the immature region of BMP-7-induced osteogenesis and bone formation. It is a powerful potential candidate to replace BMP-7 as an osteogenic stimulator for bone-related diseases.

We investigated the effect of BFP-2 on osteogenic differentiation in BMSCs and an animal model. As shown in Figure 2, we found that BFP-2 dose-dependently induced osteogenesis in BMSCs. Interestingly, BFP-2 was a more potent inducer of osteogenesis in BMSCs than BMP-7 at the same concentration. Bone-specific ALP is synthesized by osteoblasts and is presumably involved in the calcification of bone matrix.^19^ BFP-2 treatment induced ALP activity, increased expression levels of osteocalcin and CD44, and increased calcium concentration in BMSCs compared with BMP-7 treatment (Figures 3 and 4). Our current data support the hypothesis that another peptide from BMP-7 also induces osteogenesis predominantly by enhancing ALP and osteocalcin expression.

CD44 and CD51 are expressed in a variety of cell types. Recent reports have shown that CD44 and CD51 are expressed during osteogenic differentiation and can be used as biomarkers of osteogenesis. Therefore, we investigated whether BFP-2 induced CD44 and CD51 expression in BMSCs. Expression of CD44 and CD51 was measured by flow cytometry. As shown in Figure 4d, BFP-2 treatment induced CD44 and CD51 expression in BMSCs but had no effect on CD45 expression. BFP-2-treated cells were transplanted into the flanks of mice to determine whether BFP-2 induced bone formation in an animal model. Interestingly, we found that BFP-2 had potential bone-forming activity (Figure 5).

In conclusion, our study showed that the *in vitro* and *in vivo* bone-generating efficacy of BFP-2, which is derived from the immature region of BMP-7, substantially enhanced osteogenic differentiation of BMSCs and upregulated biological markers of osteogenesis. These results suggest that BFP-2 may be used as an osteogenic stimulator instead of BMP-7 in future clinical trials of bone generation engineering.
