# Supplementary material for: Bone-forming peptide-2 derived from BMP-7 enhances osteoblast differentiation from multipotent bone marrow stromal cells and bone formation
Source: Exp Mol Med. 2017 May 12;49(5):e328–. doi: 10.1038/emm.2017.40 (PMC5454442; doi:10.1038/emm.2017.40)
Supplement: Supplementary Information [file emm201740x1.docx]

**Supplemental figure legends**

**Supplemental Figure 1: BFP-1 and BFP-2 similarly induces osteogenic differentiation of BMSCs.** BMSCs were treated with 1 μg/ml of BFP-1 and 1 μg/ml of BFP-2, and assessed by Alizarin red S staining. Images are representative of three independent experiments. Magnification, x20.

**Supplemental Figure 2: Effect of ALP activity and mineralization of BFP-2 in BMSCs.** BMSCs were treated with a range of concentrations (0.01–10 μg/ml) of BFP-2 during the initial phase of osteogenic differentiation and assessed by Alizarin red S staining. The absorbance value of solubilized alizarin red was measured (A). ALP activity was quantitatively assayed by measuring the release of p-nitrophenyl phosphate with a LabAssayTM ALP Assay Kit (B).
