# Supplementary figures and images for: Bone-forming peptide-2 derived from BMP-7 enhances osteoblast differentiation from multipotent bone marrow stromal cells and bone formation
Source: Exp Mol Med. 2017 May 12;49(5):e328–. doi: 10.1038/emm.2017.40 (PMC5454442; doi:10.1038/emm.2017.40)

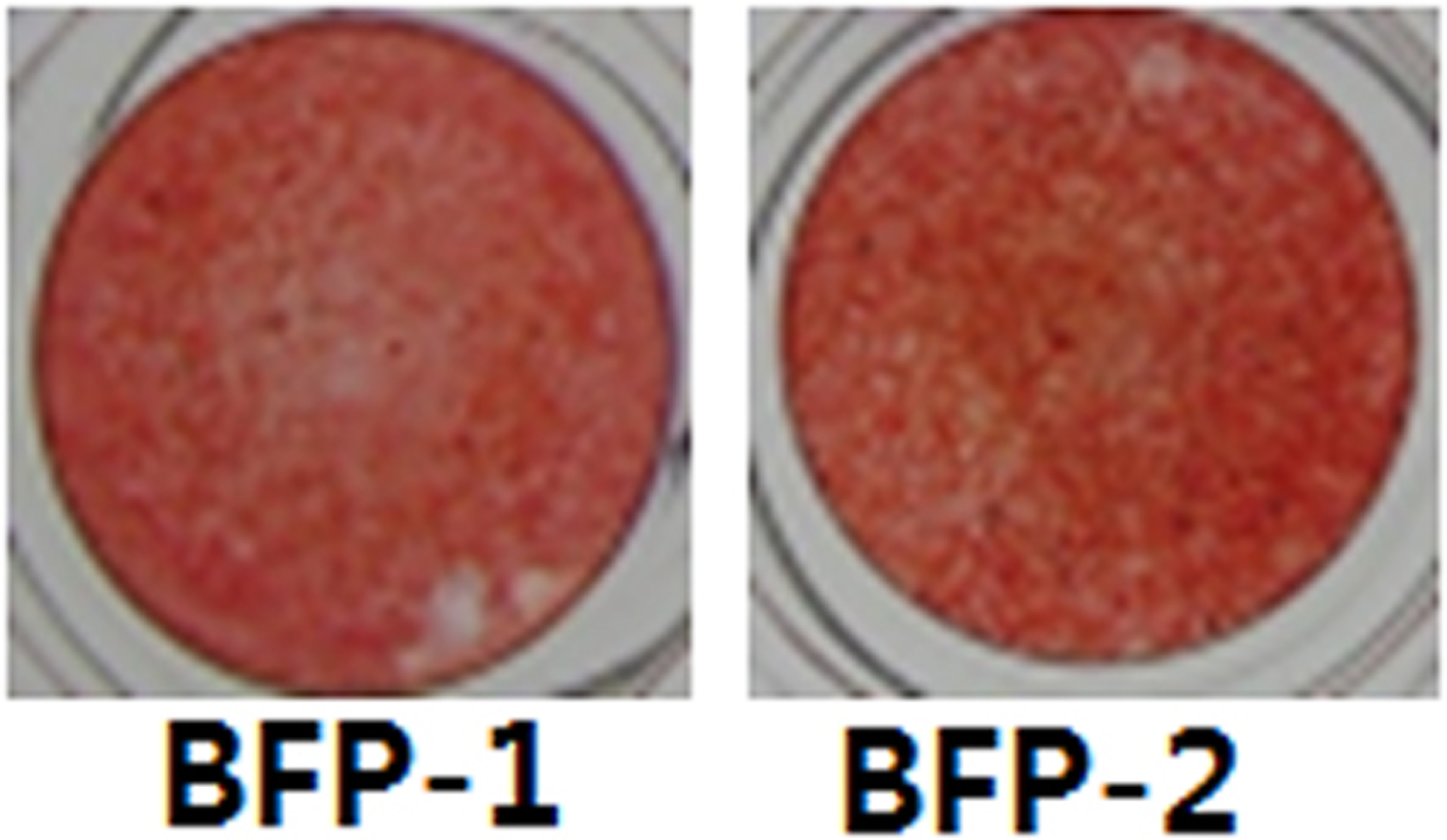

Supplement: Supplementary Figure [file emm201740x2.tif]

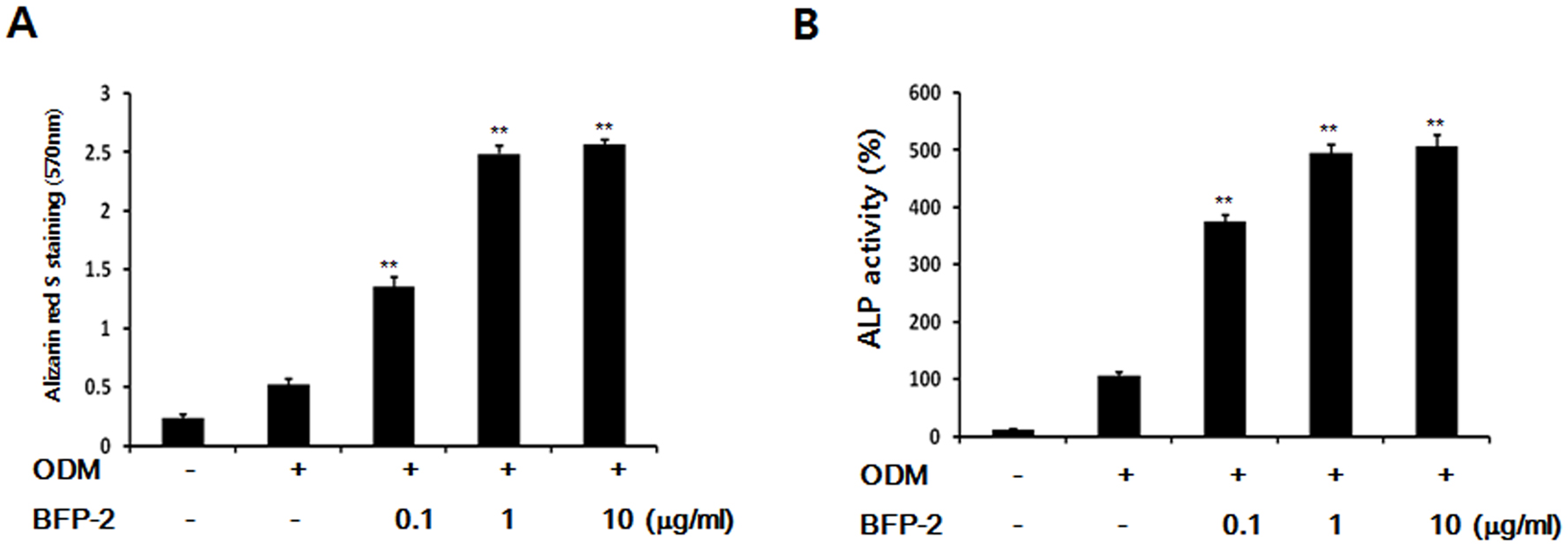

Supplement: Supplementary Figure [file emm201740x3.tif]
